# “Before I was like a Tarzan. But now, I take a pause”: mixed methods feasibility study of the Naungan Kasih parenting program to prevent violence against children in Malaysia

**DOI:** 10.1186/s12889-023-15065-4

**Published:** 2023-02-04

**Authors:** J. M. Lachman, R. Juhari, F. Stuer, P. Zinser, Q. Han, F. Gardner, A. McCoy, S. N. Yaacob, R. Kahar, M. Mansor, Z. Madon, Z. Arshat, F. Z. M. Nadzri, N. F. A. Aftar, C. Landers

**Affiliations:** 1 Department of Social Policy and Intervention, Centre for Evidence Based Intervention, University of Oxford, Oxford, England; 2 Social and Public Health Sciences Unit, University of Glasgow, Glasgow, Scotland; 3 Centre for Social Science Research, University of Cape Town, Cape Town, South Africa; 4 Faculty of Human Ecology, Universiti Putra Malaysia, Seri Kembangan, Malaysia; 5 Maestral International, Minneapolis, USA; 6 Peace Culture Foundation, Chiang Mai, Thailand; 7 Faculty of Psychology and Education, University Malaysia Sabah, Kota Kinabalu, Malaysia; 8 Mailman School of Public Health, Columbia University, New York City, USA

**Keywords:** Parenting, Child Maltreatment, Malaysia, Feasibility, Mixed-Methods

## Abstract

**Background:**

Despite impressive strides in health, social protection, and education, children continue to experience high rates of child maltreatment in Malaysia. This mixed-methods study assessed the feasibility of a five-session, social learning-based parenting program delivered by government staff in a community setting to reduce violence against children.

**Methods:**

Parents of children from birth to 17 years were recruited from two communities near Kuala Lumpur to participate in the government-run program called the Naungan Kasih Positive Parenting Program (“Protecting through Love” in Bahasa Melayu). Quantitative data from female caregivers (*N* = 74) and children ages 10–17 (*N* = 26) were collected along with qualitative interviews and focus groups with parents, children, and facilitators. The primary outcome was child maltreatment with secondary outcomes including neglect, positive parenting, acceptability of corporal punishment, harsh parenting, positive discipline, and child behavior problems. Multilevel Poisson regression and multilevel linear regression were conducted to compare baseline and post-test outcomes. Qualitative interviews and focus groups examined how participants experienced the program utilizing a thematic analysis approach.

**Results:**

Quantitative analyses found pre-post reductions in overall child maltreatment, physical abuse, emotional abuse, attitudes supporting corporal punishment, parent sense of inefficacy, and child behavior problems. There were no reported changes on positive and harsh parenting, parental mental health, and marital satisfaction, nor were there any other significant changes reported by children. Qualitative findings suggested that the program had tangible benefits for female caregivers involved in the program, with the benefits extending to their family members.

**Conclusions:**

This feasibility study is one of the few studies in Southeast Asia that examined the feasibility and initial program impact of a parenting program delivered by government staff to families with children across the developmental spectrum from birth to 17 years. Promising results suggest that the program may reduce child maltreatment across a range of child ages. Findings also indicate areas for program improvement prior to further delivery and testing, including additional training and content on sexual and reproductive health, parenting children with disabilities, and online child protection.

Despite impressive strides in health, social protection, and education in Malaysia, children continue to experience high rates of child maltreatment within the family. Recent surveys suggest that parents and caregivers account for over 40% of all reported abuse and neglect cases, with over 75% of children experiencing some form of child maltreatment and 53% experiencing parental physical maltreatment [1, 2]. Malaysian government authorities and children’s rights advocates, such as UNICEF, have also highlighted a number of child protection issues that require addressing, including child marriage, online safety, gender-based violence, and the vulnerabilities of children with disabilities and children of refugee and migrant families [3].

Research over the past several decades shows that the scale-up of parenting programs may be an effective strategy to reduce violence against children and improving child wellbeing [4]. Recent systematic reviews have identified a growing body of literature supporting the effectiveness of parenting programs in low- and middle-income countries [5–8], with 130 identified trials in a recent systematic review [9]. A recent systematic review of 11 rigorous studies on parenting programs in East Asia and the Pacific region illustrates the positive effects of reducing abusive, harsh, or negative parenting and improving positive parenting [10]. However, many tested interventions focus on a specific age range with very few that cover the entire child development spectrum from birth to 17 years.

Consequently, positive parenting interventions have been identified as a critical strategy within THRIVES, the Centers for Disease Control and Prevention's technical package to prevent VAC and the World Health Organization’s INSPIRE Framework [11, 12]. Interventions that support the development of safe, stable, and nurturing relationships between parents or other primary caregivers and their children are a key evidence‐based strategy for violence prevention for three reasons [13, 14]. First, they can prevent violence towards children; second, they may also prevent the early development of violent behavior in children; and third, they may also prevent perpetration and victimization of violence during adulthood. Emerging evidence suggests that by stemming the early development of violent behavior, such relationships can also reduce many types of violence occurring in adolescence and early adulthood, such as youth violence, dating violence, sexual violence, and self‐directed violence [15].

Emerging research suggesting that parenting programs may be as effective when tested in new contexts as they were in their country of origin supports the case for transporting programs from one context to another [16]. Importing interventions and culturally adapting them as opposed to creating brand new ones has various advantages, such as the process for their development is less time-consuming and more cost-effective. The key to success is selecting the 'source' parenting intervention because of its evidence base and then adapting the imported intervention in a culturally appropriate way to ensure parenting messages resonate with the target audience [17]. It is also important that these programs are low-cost and can be adapted to fit local contexts without restrictive licensing fees or constraints. The Parenting for Lifelong Health programs for caregivers of young children and adolescents (PLH) and WHO and UNICEF-developed Care for Child’s Healthy Growth and Development (CCD) program for caregivers of children ages zero to three years [18, 19] are examples of such ‘source’ programs that are freely-available and have been widely disseminated in low-resource setting throughout the Global South.

In Malaysia, Lembaga Penduduk & Pembangunan Keluarga Negara (National Population and Family Development Board, or LPPKN) in the Ministry of Women, Family and Community Development is the primary government agency responsible for family strengthening. LPPKN’s portfolio of family programs is focused on reproductive health, population, and family development education. As part of an effort to strengthen the evidence base of LPPKN’s existing core programs, the Naungan Kasih Positive Parenting Program (“Naungan Kasih” means “Protection through Love” in Bahasa Melayu) was developed by combining local Malaysian program content with content from the PLH and CCD programs to create a universal program for caregivers of children from birth to 17 years that was grounded in evidence-based content and rooted in local cultures and customs. This study aimed to test the feasibility of the Naungan Kasih program by examining its initial impact on reducing VAC and associated risk factors. It also qualitatively examined program implementation fidelity and quality; recruitment, retention, and engagement of participants; and cultural and contextual relevance, acceptability, and satisfaction.

## Methods

### Design

This pre-post study was conducted in two urban communities close to Kuala Lumpur, Malaysia – Shah Alam and Putrajaya – from November 2018 to April 2019. Ethical Procedures were approved by the University of Oxford Central University Research Ethics Committee (Ref: R0655_RE002) and Universiti Putra Malaysia (Ref: UPM/TNCPI/RMC/1.4.18.2).

### Participants

Parents and caregivers (*N* = 74) were recruited in November 2019 via telephone calls, letters, or home/community visits using a targeted sampling approach based on referrals from LPKKN for families requiring support managing difficult child behavior or requesting parenting advice. Families were drawn from two communities close to Kuala Lumpur, one from a low-income area of Shah Alam, and one from mixed low- and middle-income areas of Putrajaya. Adult participants had to provide written informed consent, be age 18 or older, and be a primary caregiver responsible for the care of a child who resides in the same household for at least four nights a week in the previous month. An additional 26 children aged 10 to 17 years were recruited for quantitative and qualitative interviews. Eligible children were those whose parents participated in the study and lived in the same house as their parents at least four nights per week. Parents were asked to identify one child to report on based on the child they were having the most difficulties with in their family.

Participants were given a food voucher, parcel, or cash incentive (approximately 5 USD per participant) after each assessment at baseline and post-test as well as after Focus Group Discussions (FGDs) and interviews. Refreshments (i.e., juice/soda, tea, sugar, and bread) were provided during data collection, and all participants received a certificate on concluding the study. Participants also received a nutritious lunch at every group parenting session and a certificate of completion at the end of the program. Any participant who chose to leave the study early received a certificate confirming the number of sessions attended.

### Sample size

The sample size was determined based on a maximum possible sample within the funding constraints.

### Training of facilitators and coaches

Training of Naungan Kasih facilitators was conducted in early January 2019 over an intensive 4-day workshop in Kuala Lumpur. Twenty-five staff members from LPPKN and five researchers from the University of Putra Malaysia (UPM) participated in the training. LPPKN staff included personnel responsible for communications, counselling, community development, and statistics. At the end of the training, 12 staff members who were based in Putrajaya and Kuala Lumpur were selected to deliver the program during the feasibility pilot. These facilitators were 83% female (*n* = 10), 83% married, with ages ranging from 32 to 56 years. The facilitators had a minimum qualification degree in communications, counselling, or community development. All but two facilitators were parents themselves.

A further two-day supervision training workshop was provided to six staff members from the Universiti Putra Malaysia Faculty of Family Ecology who had also participated in the facilitator training workshop and contributed to the program development. They conducted five weekly supervision sessions lasting approximately two hours each with facilitators during program delivery. These sessions focused on troubleshooting challenges experienced during program delivery using participatory methods that included group discussions, role-plays, and joint problem solving.

### Intervention

Naungan Kasih is a group-based family strengthening program for families with children from birth to 17 years. The overall goal is to promote child wellbeing by helping parents develop the skills to establish positive relationships with their children while reducing the risk of violence against children at home and in their communities. It was developed in collaboration with the Malaysian government by integrating the main LPPKN parenting programs – the Belaian Kasih (for parents of children up to 13 years of age) and Mutiara Kasih programs (14 to 17 years) – with content from the Parenting for Lifelong Health (PLH) programs for young children and adolescents [20, 21], and the WHO and UNICEF’s Care for Child’s Healthy Growth and Development [19].

The Parenting for Lifelong Health program for young children was originally developed by integrating common evidence-based program components within the local cultural context in South Africa [21]. Subsequent testing in randomized controlled trials in South Africa, Thailand, and Philippines demonstrated positive impacts on reduced violence against children and child behavior problems, improved caregiver mental health and positive parenting, and reduced intimate partner violence, the latter in the Philippines [22, 23]. Similarly, the Parenting for Lifelong Health program for parents and adolescents was developed in rural South Africa and then tested in a cluster randomized controlled trial showing positive effects across a range of family outcomes [20]. Since their initial development in South Africa, PLH programs have been adapted and delivered in over 30 countries [24]. The Care for Child’s Healthy Growth and Development (CCD) was developed in 2002 by UNICEF and WHO support caregiver involvement in early child development through responsive nurturing care [19]. CCD has been delivered and tested in multiple countries, with results randomized trials demonstrating improved parenting practices, reduced harsh punishment, and increased cognitive development [25].

**Fig. 1:**
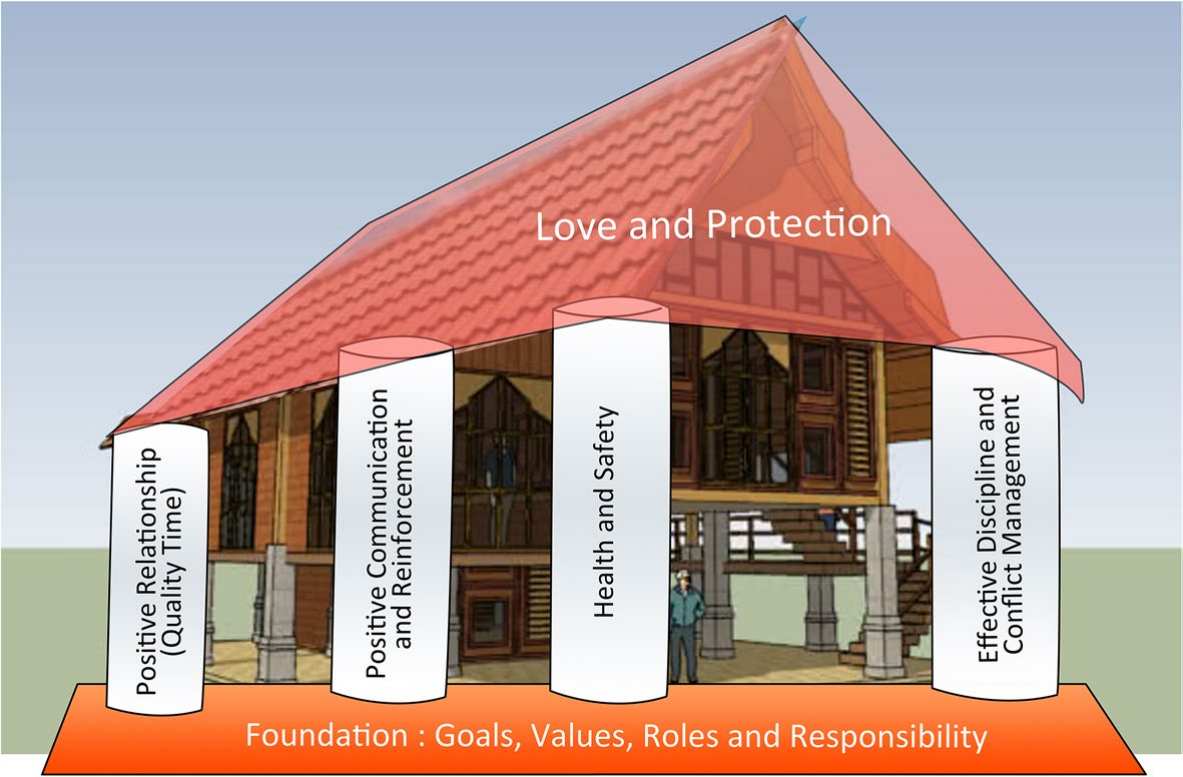
Conceptual model for the Naungan Kasih program

The subsequently developed Naungan Kasih program was designed as a universal program for parents and caregivers of children from birth to 17 years. Using a metaphor of building a house of support for parents and their children, the program was based on social learning and attachment theories. Topics included the following core themes delivered over six weekly sessions lasting approximately two hours each: 1) Establishing parenting goals, values, roles, and responsibilities, 2) Positive relationship building through quality time, 3) Positive communication and reinforcement, 4) Health and safety through rules and routines, 5) Effective discipline and conflict management, including content on addressing sexual violence disclosure by adolescents, and 6) Reflection and celebration as an end to the program (See Fig. 1).

Participants were divided into groups according to the age of their target child (i.e., birth-23 months, 2–9 years, or 10–17 years). Each group was delivered by two trained facilitators who delivered specific program modules designed to support parents and caregivers of the targeted age range. They used a participatory active learning approach to engage parents that included a combination of group discussions, illustrated comics demonstrating parenting skills, role-plays, and assigned home activities. Caregivers also had the opportunity to share feedback on their experiences applying new parenting skills at the beginning of each subsequent session. Childcare was provided for caregivers who required it, and each session ended with a lunch.

### Data collection procedures

Data collection during the pilot took place at two assessment points: baseline in December 2018 and post-test in April 2019 with the program delivered from mid-January 2018 to the end of February 2019 (i.e., post-test as four months post-baseline and one-month post-intervention). Participants were assessed in the same order at baseline and post-test to maintain similar timing in between assessments.

Data collectors fluent in Bahasa Melayu were recruited from University Putra Malaysia. All had previous experience working with vulnerable families and received 20 h of training. Interview guides and procedures for each data collection point were scripted to promote consistency across data collectors. All measures and tools were translated from English into Bahasa Melayu, and the translations were checked by back-translations.

Participants were assigned a study ID following receipt of informed consent. We used Computer-Assisted Self-Interviewing ('CASI') methods with e-tablet technology to administer consent forms, screening, outcome and satisfaction questionnaires, and observational assessments. Audio-CASI was used to administer sensitive items on the questionnaires regarding child maltreatment. Participants received instruction on how to use the e-tablets through tutorials prior to administering questionnaires. Completion of the questionnaires took 60–90 min.

Qualitative data collection occurred after program delivery. Three FGDs with adult participants (*n* = 7–11 per FGD) and one FGD with adolescents (*n* = 12) were conducted in community centers and lasted approximately 120 min. Nine in-depth adult interviews lasting approximately 60 min were conducted in the community centers or in participant homes with selected targeting based on levels of program attendance (*n* = 4 with those who attended 4 or more sessions, *n* = 3 for those who attended 2 or fewer sessions, and *n* = 2 for those with no attendance). To further engage adolescents in FGDs, they were asked to draw pictures depicting their relationship with their parents/primary caregivers and to share whether this had changed during the program. FGDs were conducted with all 12 of the LPPKN facilitators who delivered the Naungan Kasih program. Ten were female and two were male, ages ranged from 32 to 56 years old, and 83.3% were parents themselves (*n* = 10). Eight facilitators (75%) also had over ten years of experience working with LPPKN. FGDs and interviews were captured on digital recorders with written notes as backup. Data was transcribed verbatim and was translated into English and back translated into local languages to verify accuracy.

### Outcome measures

Standardized measurements, multiple sources (i.e., self-reports by parents/primary caregivers and children), and standard timeframe of events or practices over the 'past month' were used to strengthen study validity and identify potential changes in behavior due to the intervention.

#### Family demographics

Baseline adult demographics included age, gender, language, ethnicity, citizenship, religion, relationship to head of household, marital status, education, physical health, disabilities, and literacy level. We also assessed caregivers’ relationship with target child, number of children in their care, and child age and sex. Adult history of experiencing child maltreatment was measured through an adapted version of the International Society for the Prevention of Child Abuse and Neglect (ISPCAN) Child Abuse Screening Tools Retrospective version (ICAST-R) (4 items) [26]. Socioeconomic factors included adult employment and adult-report of relative poverty based on five items from the Multiple Indicator Cluster Survey (MICS) [27].

#### Primary outcome—child maltreatment

Child maltreatment was measured using 26 items from an adapted and expanded version of the ISPCAN Child Abuse Screening Tool-Trial Caregiver and Teen version (ICAST-T) [28]. The ICAST-T measures adult- and child-report of the incidence of abuse perpetrated against their child over the past month using a frequency score on a scale of 0 to 7, or 8 or more times (e.g., "In the past 4 weeks, how often did you discipline [Child Nickname] by pushing, grabbing, or kicking him/her?"). This study assessed the incidence of physical abuse (13 items), emotional abuse (7 items), and neglect (6 items), as well as the frequency of overall abuse by summing the physical and emotional abuse subscales.

#### Secondary outcomes

Adult- and child-report secondary outcomes included parenting behavior assessed using the Alabama Parenting Questionnaire – Parent/Child Report (APQ, 42 items with an additional item on supervision of social media use) [29] and endorsement of corporal punishment using one item from the MICS Child Discipline module [27]. Child externalizing problems (33 items) and child internalizing problems (21 items) were assessed using the Child Behavior Checklist (CBCL) for families with children ages 2 to 17 years [30]. We also assessed adult-report of parental mental health problems with the Depression, Anxiety, and Stress Scale short form (DASS-21; 21 items) [31] and adult-report of sense of inefficacy in ability to manage child behavior problems (ICAST-Efficacy; 2 items) [28].

#### Process evaluation measurements

Implementation fidelity was measured via self-report checklists by facilitators. The self-report checklist gauged the level of adherence to the activities described in the facilitator manual for each module. The sessions were also monitored by trained supervisors from Universiti Putra Malaysia who noted any challenges with program delivery where were discussed during supervision sessions.

Program adherence by participants was assessed by examining rates of enrolment, attendance, dropout, completion, and engagement of home activities. Enrolment rates were based on the ratio of those completing baseline assessments to those who attend at least one session. Mean attendance rates for enrolled participants were determined based on the ratio of the number of attended sessions to the total number of five program sessions. Dropout rates for enrolled participants were defined as the percentage of participants who fail to attend at least three consecutive sessions and did not attend any sessions at a later stage. Completion rates for the entire allocation group were determined based on the number of enrolled participants who attended a cut-off threshold of at least 66% of the program.

Satisfaction, acceptability, and engagement of the program (45 items) was assessed at post-intervention and examined perceptions of program satisfaction, acceptability of delivery and teaching methods, acceptability of theoretical parenting techniques, and appropriateness of program facilitators.

### Analytic methods

**Fig. 2:**
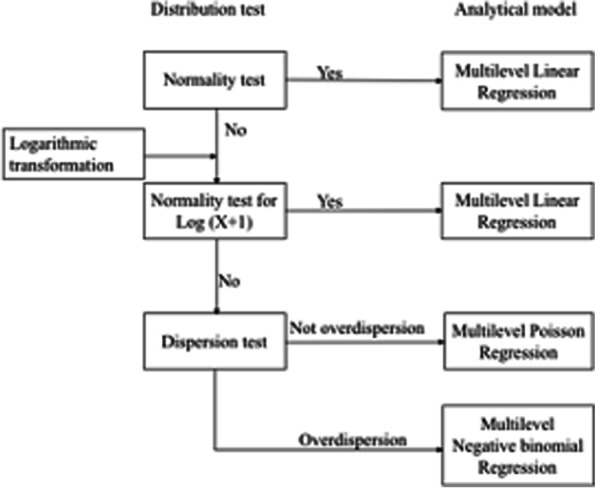
Distribution test flowchart

Baseline characteristics of Shah Alam and Putrajaya groups were examined along with differences between groups based on Chi-squared or independent T-test analyses. For all outcomes with a frequency as the unit of measure, a distribution test was first performed to determine the appropriate type of regression (see Fig. 2). For outcomes that measure as continuous variables, multilevel linear regression with participants as a random effect was used to test the baseline and post-test differences (participant is the random effect). As Table 1 indicates, all maltreatment outcomes failed normality tests even after log transformations (*p* < 0.001) but were not overly dispersed based on dispersion tests (*p* > 0.05). As a result, multilevel Poisson regressions were conducted for these outcomes to compare baseline and post-test outcomes with participant as a random effect.

**Table 1:** Distribution check for maltreatment outcomes based on adult-report

Outcome^a^	Normality test		Normality test after log transformation		Dispersion test	
	**W**	***p* value**	**W**	***p* value**	**Chi-sq**	***p* value**
Overall child maltreatment	0.77	< 0.001	0.90	< 0.001	117.15	0.864
Physical abuse	0.54	< 0.001	0.63	< 0.001	48.84	1.000
Emotional abuse	0.78	< 0.001	0.90	< 0.001	109.84	0.945
Neglect	0.29	< 0.001	0.38	< 0.001	24.04	1.000
Corporal punishment	0.63	< 0.001	0.63	< 0.001	77.00	1.000

^a^ Based on ISPCAN Child Abuse Screening Tool-Trial

### Qualitative data analysis

Qualitative data analysis examined the views and experiences of participants, facilitators, and staff members using a thematic analysis approach with open, axial, and selective coding [32]. Two independent raters assessed and coded data from FGDs and interview transcripts. The study investigated emergent themes regarding 1) the changes participants observe in their parenting practices and child behavior at home during the program; 2) acceptability and appropriateness of program materials, delivery, and key components; and 3) existing barriers to participation during sessions and engagement in home practice and other activities. Interviews explored the challenges experienced by facilitators in implementing the program on both a logistical level (e.g., recruitment, session length, location, meals) and process level (e.g., using a collaborative approach and/or explaining concepts such as child-led play).

## Results

### Sample characteristics at baseline

**Fig. 3:**
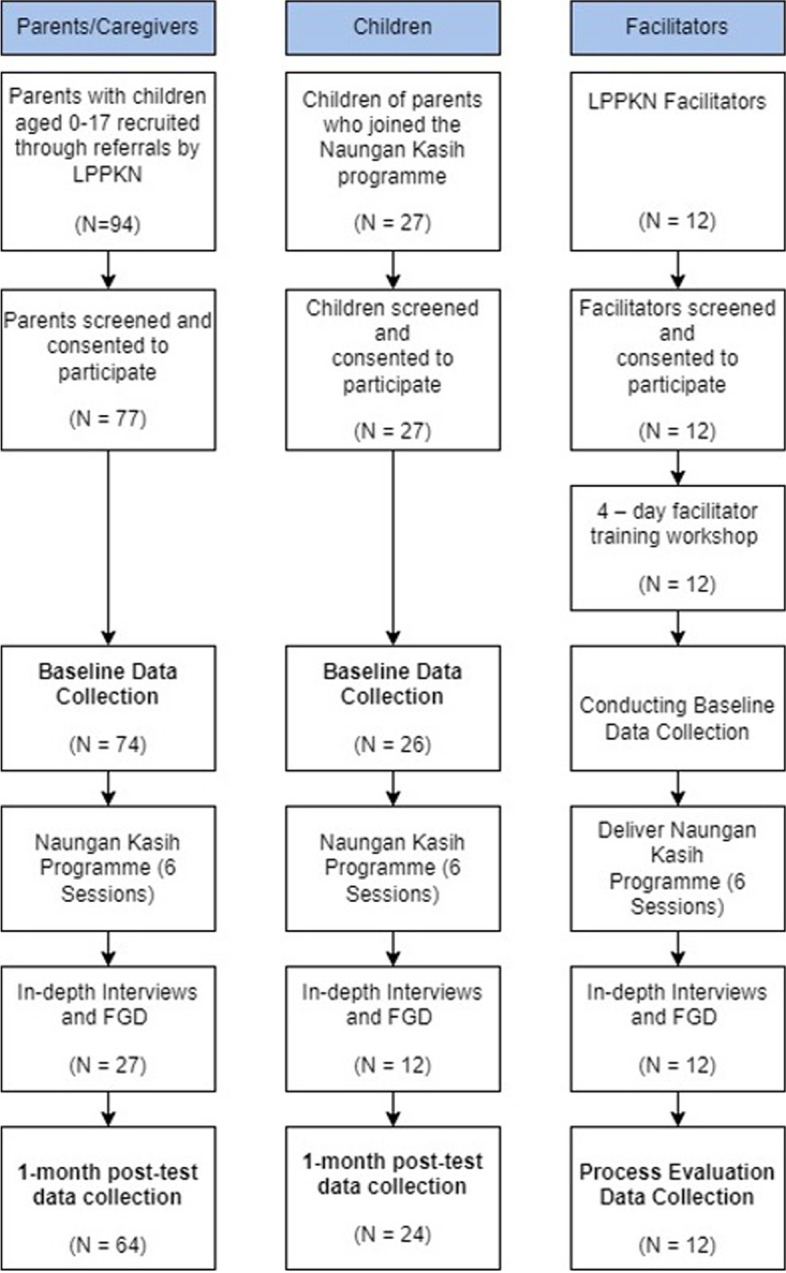
Participant flow

A summary of participation in this study is provided in Fig. 3. The overwhelming majority of adult participants were female and of Malay ethnicity. Parents were on average around 43 years old (M = 42.55, SD 9.37). Twenty parents selected children between the ages of birth to 23 months (27.0%), 28 parents selected children ages two to nine years (37.8%), and 24 parents selected children ages ten to 17 years (35.1%).

Overall, parents in Putrajaya reported a higher socioeconomic status than those from Shah Alam. Although three-quarters of the total sample (*n* = 67, 75.6%) had no university schooling, Putrajaya parents (*n* = 18, 54.5%) were more educated than those in Shah Alam (*n* = 0, 0.0%; Chi-squared = 29.55, *p* < 0.001), and less likely to be receiving government financial assistance (Chi-squared = 6.06, *p* = 0.014). Likewise, although 90.5% of the parents were married (*n* = 67), 100% of the female caregivers were married parents in Putrajaya in comparison to Shah Alam where 82.9% were married (*n* = 34, Chi-squared = 2.22, *p* = 0.045). Parents in Putrajaya were also more likely to be employed (*n* = 23, 69.7% vs *n* = 10, 24.4%; Chi-squared = 15.10, *p* < 0.001) than those in Shah Alam (see Table 2).

**Table 2:** Characteristics of adult participants at baseline

	**Total** **(*N* = 74)**	**Shah Alam** **(*N* = 41)**	**Putrajaya** **(*N* = 33)**
**Adult demographics**			
Age, M (SD)	42.55 (9.37)	46.41 (9.88)***	37.76 (5.94)***
Gender: Female, *n* (%)	72 (97.3)	41 (100.0)	31 (93.9)
Ethnicity: Malay, *n* (%)	72 (97.3)	40 (97.6)	33 (97.0)
Education: University schooling, *n* (%)	18 (24.3)	0 (0.0)***	18 (54.5)***
Literacy: Can read easily, *n* (%)	73 (98.6)	40 (97.6)	33 (100.0)
Marital status: Married, *n* (%)	67 (90.5)	34 (82.9)*	33 (100.0)*
Wife of head of household, *n* (%)	63 (85.1)	33 (80.5)	30 (90.9)
Adult disability, *n* (%)	8 (10.8)	3 (7.3)	5 (15.2)
Experienced maltreatment during childhood, *n* (%)	41 (55.4)	19 (46.3)	22 (66.7)
**Target child demographics**			
Birth to 23 months, *n* (%)	20 (27.0)	10 (24.4)	10 (30.3)
2 to 9 years, *n* (%)	28 (37.8)	15 (36.6)	13 (39.4)
10 to 17 years, *n* (%)	26 (35.1)	16 (39.0)	10 (30.3)
Age, M (SD)	7.32 (5.35)	7.84 (5.86)	6.67 (4.64)
Gender: Female, *n* (%)	44 (59.5)	26 (63.4)	18 (54.5)
Child biological son/daughter, *n* (%)	59 (79.7)	30 (73.2)	29 (87.9)
**Family demographics**			
Household size, M (SD)	5.24 (1.45)	5.12 (1.63)	5.39 (1.20)
Presence of another caregiver, *n* (%)	36 (48.6)	24 (58.5)	12 (36.2)
Parent employed, *n* (%)	33 (44.6)	10 (24.4)***	23 (69.7)***
Other adult employed, *n* (%)	62 (83.8)	30 (73.2)**	32 (97.0)**
Family receives government support, *n* (%)	22 (29.7)	17 (41.5)*	5 (15.2)*
Household assets, M (SD)^a^	8.14 (1.93)	7.39 (1.51)***	9.06 (2.01)***

^*^ *p* < .05

^**^ *p* < .01

^***^ *p* < .001

^a^ MICS Survey; Significant differences between Shah Alam and Putrajaya groups based on Chi-squared or Independent T-test analyses

Twenty-six adolescents were recruited from enrolled families to participate in qualitative focus groups. The mean age was 14 years with child respondents in Shah Alam (M = 14.69, SD 1.92) significantly older than those in Putrajaya (M = 12.90, SD 2.13; *F*(1,25) = 6.59, *p* = 0.017). Two-thirds of the adolescent respondents were female (*n* = 17, 65.4%) and most of them were enrolled in school (*n* = 25, 96.2%; see Table 3).

**Table 3:** Characteristics of adolescent respondents at baseline

	**Total** **(*N* = 26)**	**Shah Alam** **(*N* = 16)**	**Putrajaya** **(*N* = 10)**
Age, M (SD)^a^	14.00 (2.15)	14.69 (1.92)*	12.90 (2.13)*
Gender: Female, *n* (%)	17 (65.4)	11 (68.8)	6 (60.0)
Enrolled in school, *n* (%)	25 (96.2)	15 (93.8)	10 (100.0)

^a^ Significant differences between Shah Alam and Putrajaya groups based on Chi-squared or Independent T-test analyses

^*^ *p* < .05

### Quantitative results

#### Program engagement and fidelity

Sixty-three out of 74 parents attended at least one session of the program (85.1%) with an overall attendance rate of 68.2% or 4.1 out of 6 sessions. Forty-five parents attended five or more sessions (60.8%), with 55 parents (74.3%) attending at least two-thirds of the program (i.e., four or more sessions). Comparing enrolment and attendance across implementation sites, parents in Shah Alam had a higher enrolment rate (Shah Alam: 92.7%; Putrajaya: 75.8%; *X*^2^ (1, 74) = 4.14, *p* = 0.042) and attendance rate (Shah Alam: 76.0%; Putrajaya: 58.6%; *F*_1,71_ = 4.77; *p* = 0.032) than those in Putrajaya. Associations with higher rates of attendance included parents with older children (*r* = 0.267, *p* = 0.036) and unemployed parents (*r* = 0.429, *p* < 0.001), potentially due to availability to attend group sessions during working days.

Self-report fidelity checklists indicated that the facilitators delivered over 90% of activities across the six sessions, which were corroborated by assessments by supervisors who observed sessions.

#### Primary outcome: child maltreatment

**Fig. 4:**
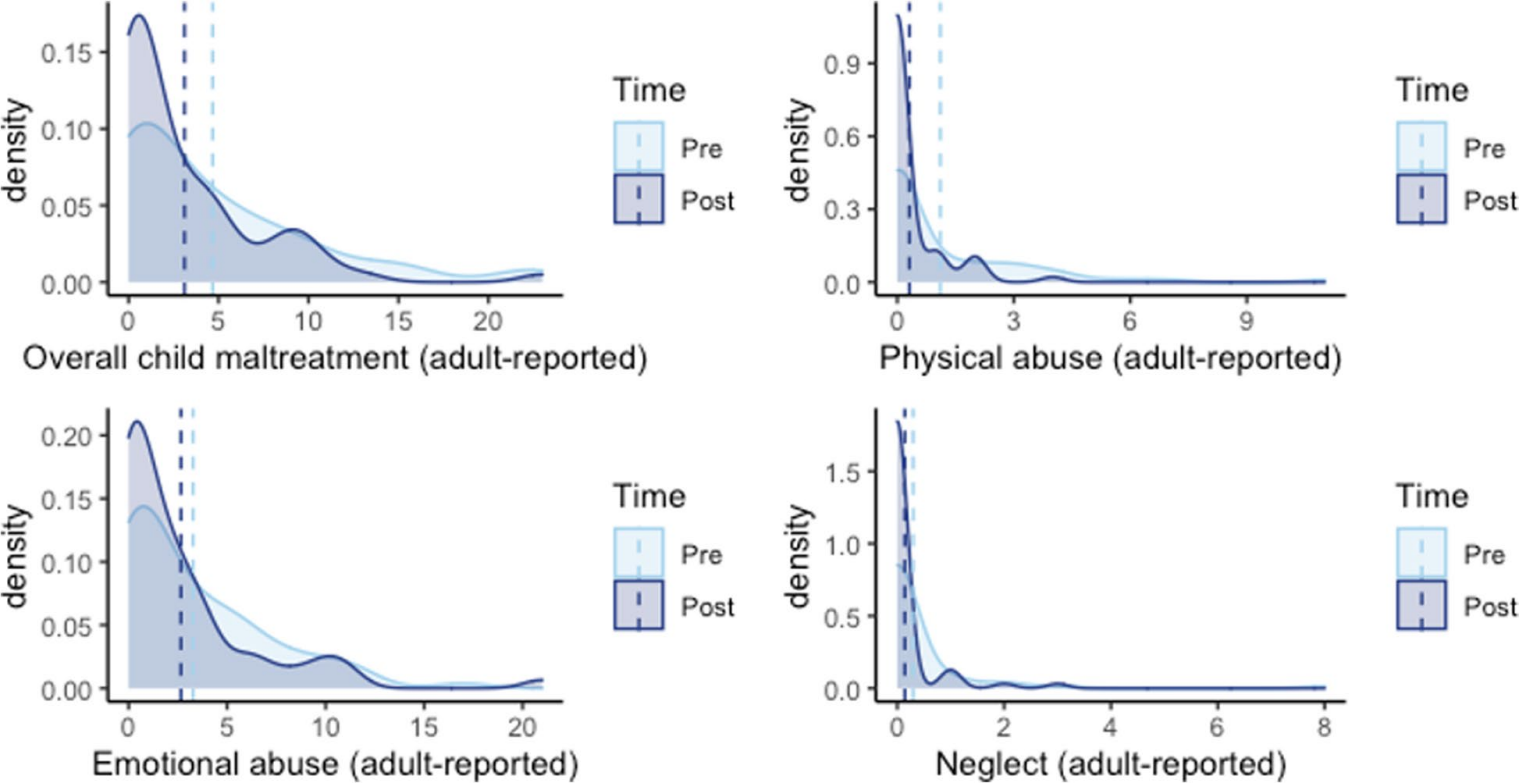
Child maltreatment outcomes at baseline and post-test based on adult-report

**Fig. 5:**
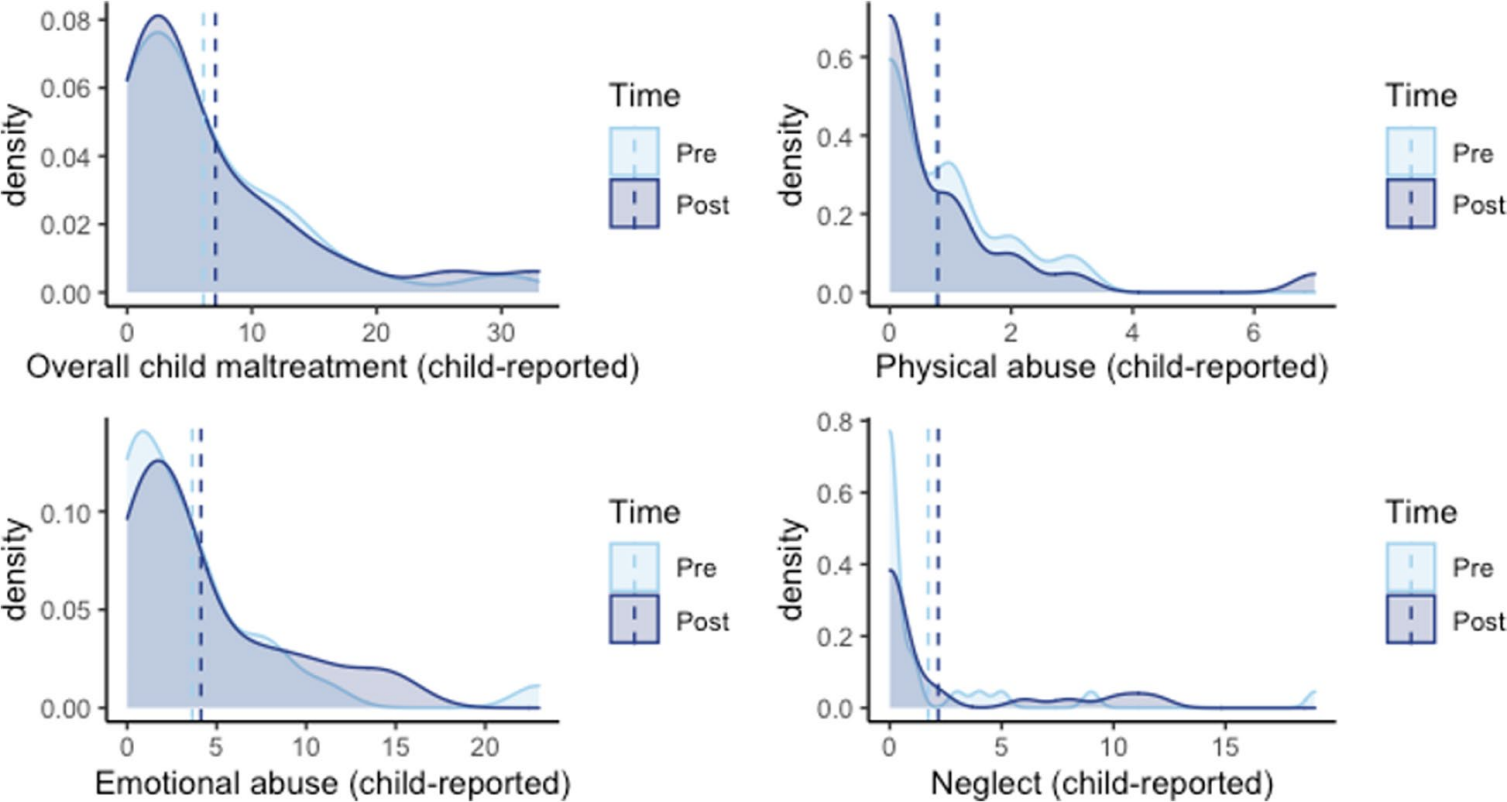
The maltreatment outcomes at both baseline and post-test based on child-report

All the comparisons for child maltreatment outcomes between baseline and post-test were conducted by multilevel Poisson regression. Parents reported 32% decreased overall abuse at post-test (IRR = 0.68 [0.57, 0.81]), 71% reduced physical abuse (IRR = 0.29 [0.18, 0.47]), and 19% reduced emotional abuse (IRR = 0.81 [0.66, 0.99]). There were no differences between baseline and post-test on the frequencies of parent-reported child neglect nor any differences on the frequencies of child maltreatment reported by the smaller sample of child respondents (see Tables 4 and 5 and Figs. 4 and 5).

**Table 4:** Results comparing adult-reported maltreatment outcomes at baseline and post-test (*n* = 74)

Outcome^a^	Baseline		Post-text		*P* value	IRR	95% lower CI	95% upper CI
	**Mean**	**SD**	**Mean**	**SD**				
Overall Abuse	4.68	5.62	3.11	4.22	< 0.001***	0.68	0.57	0.81
Physical Abuse	1.11	1.99	0.31	0.75	< 0.001***	0.29	0.18	0.47
Emotional Abuse	3.27	3.71	2.66	3.80	0.036*	0.81	0.66	0.99
Neglect	0.30	1.07	0.14	0.50	0.430	0.71	0.30	1.66

^*^ *p* < .05

^***^ *p* < .001

^a^ Adult-report of ICAST-Trial

**Table 5:** Results comparing child-reported maltreatment outcomes at baseline and post-test (*n* = 26)

Outcome^a^	Baseline		Post-text		*P* value	IRR	95% lower CI	95% upper CI
	**Mean**	**SD**	**Mean**	**SD**				
Overall Child Maltreatment	6.12	7.10	7.08	8.42	0.261	1.14	0.91	1.42
Physical Abuse	0.76	0.97	0.79	1.56	0.900	1.04	0.55	1.98
Emotional Abuse	3.64	5.03	4.13	4.44	0.868	1.02	0.77	1.37
Neglect	1.72	4.19	2.17	3.96	0.071	1.49	0.97	2.29

^a^ Child-report of ICAST-Trial

#### Secondary outcomes

Adults reported a 74% reduction in endorsement of corporal punishment (IRR = 0.26 [0.09, 0.75]). In addition, adults reported reduced overall child behavior problems (*β* = -2.19; *p* = 0.23) and reduced sense of parenting inefficacy (*β* = -0.43; *p* = 0.004) for their children ages 6 to 17 years. There were no other significant differences between baseline and post-test for parent-report of responsivity, involvement, positive discipline, parent mental health problems, and marital satisfaction (Table 6). No significant differences were found for secondary outcomes based on child report (Table 7).

**Table 6:** Results comparing secondary outcomes at baseline and post-test based on adult-report

Outcome	N	Baseline		Post-test		*β*	P value	IRR^a^	95% lower CI	95% upper CI
		**Mean**	**SD**	**Mean**	**SD**					
Positive parenting (early childhood)^b^	20	26.95	2.48	27.67	2.09	0.87	0.171	–-	-0.32	2.07
Responsivity^b^	20	7.15	0.81	7.47	0.64	0.33	0.092	–-	-0.03	0.68
Harsh parenting (early childhood)^b^	20	0.80	1.24	0.93	1.22	0.18	0.507	–-	-0.33	0.69
Involvement^b^	20	5.30	0.66	5.73	0.59	0.43	0.053	–-	0.00	0.86
Positive parenting^c^	54	42.06	4.44	41.57	6.44	-0.48	0.544	–-	-2.01	1.05
Harsh parenting^c^	54	7.17	6.08	6.55	5.09	-0.37	0.534	–-	-1.54	0.79
Positive discipline^c^	54	3.13	1.78	3.61	1.97	0.47	0.137	–-	-0.14	1.09
Child total behavior problems^d^	54	13.85	10.57	11.30	8.31	-2.19	0.023*	–-	-4.01	-0.37
Parental mental health problems^e^	74	5.31	6.04	5.17	5.42	-0.29	0.712	–-	-1.82	1.24
Marital satisfaction^f^	74	17.79	3.11	17.47	2.35	-0.10	0.707	–-	-0.65	0.44
Attitudes supporting corporal punishment^g^	74	0.53	0.50	0.34	0.48	-1.36	0.012*	0.26	0.09	0.75
Parent sense of inefficacy^h^	74	2.70	1.30	2.25	0.98	-0.43	0.004**	–-	-0.72	-0.14

^*^ *p* < .05

^**^ *p* < .01

^a^ IRR only conducted for outcomes assessed using multilevel Poisson regressions. All other outcomes assessed using multilevel linear regressions

^b^ HOME Inventory interview items for parents of children ages birth-23 months

^c^ Alabama Parenting Questionnaire for parents of children ages 0 to 17 years

^d^Child Behavior Checklist for parents of children ages 2–17 years

^e^ Depression Anxiety and Stress Scale

^f^ Kansas Marital Satisfaction Survey

^g^ MICS Survey

^h^ ICAST Parent Inefficacy subscale

**Table 7:** Results comparing secondary outcomes at baseline and post-test based on child-report

Outcomes	N	Baseline		Post-test		*β*	*P* value	IRR^a^	95% lower CI	95% upper CI
		**Mean**	**SD**	**Mean**	**SD**					
Positive parenting^b^	26	35.08	9.73	35.50	9.25	-0.04	0.980	–-	-0.03	3.31
Harsh parenting^b^	26	16.68	5.90	17.33	5.21	0.20	0.860	–-	-3.40	2.37
Positive discipline^b^	26	3.28	1.59	3.17	1.46	-0.17	0.601	–-	-1.97	0.45
Child total behavior problems^c^	26	17.80	9.73	19.75	10.70	1.16	0.426	–-	-0.78	3.97
Corporal punishment^b^	26	0.28	0.46	0.21	0.41	-0.29	0.614	0.74	0.23	2.34

^a^ IRR only conducted for outcomes assessed using multilevel Poisson regressions. All other outcomes assessed using multilevel linear regressions

^b^ Alabama Parenting Questionnaire

^c^ Child Behavior Checklist

## Qualitative Results

### Adult respondents

#### General feelings about participation in the LPPKN parenting program

In general, the program was well received and accepted by parents. Many parents reported perceived benefits of the program on improving their relationship with their children: "When I joined the program, it was fun for me, I feel happy that I can apply things that I learned with my kids, on discipline, on communication with them" (44 years old, infant group). Parents often commented on the benefits of small group format of delivery that allowed for individual attention and participation, which was different than other programs they had attended in the past: "I'm excited, we do [it] in small groups. Not as usual program, now we can focus. In small groups, facilitators can give more attention to each participant…. We can understand the content better. When we ask questions, there will be people who respond” (27 years old, infant group). Parents also shared that they enjoyed attending the program as “day out” or “me time” to meet with friends and learn new things: “Like me, I am not working, so when I got to go out on Saturdays for this program, it felt like it is an outing day for me, a day for me to get out and meet with friends, learn new things… It is my time” (47 years old, adolescent group).

#### Changes in the way parents discipline their children

Most respondents reported that Naungan Kasih helped reduce their tendency to use physical punishment. As one mother offered, “Before, I used to cane my child when I couldn’t control them. Now, I do not do that anymore” (35 years old, child group). They also shared that they were less likely to scold or verbally abuse their children. As one parent explained, this was directly connected to their ability to be less reactive to their children by using the “Taking a Pause” technique.

It is like this … my children say that before I always scold them … but now it is different … there are less anger and scolding. Before I was like a Tarzan. But now … when I feel angry, I take a pause. And the children said, Mum, your voice is not like a Tarzan anymore. There are changes like that. Whenever I feel angry, I took a pause. Even though the positive changes are not 100%, but there are some effects. (32 years old, infant group)

In addition, some parents noticed changes in their discipline style, especially in their ability in establishing household rules and using the technique “When you do this, then you can do that” to increase compliance. As one mother explained, “There are some changes in discipline style. For example, if the child wants to go out and play, I will allow them to do so, with the condition – only after they have completed their schoolwork. After taking a shower, my child knows where to put their used clothing” (53 years old, child group).

#### Changes in parents’ own life

Many parents noticed positive changes in their own lives and their relationships with their partners. For instance, one mother noticed that her improved parenting efficacy also reduced stress for herself: “I experience less stress with the child because now I know how to communicate with her” (27 years old, infant group). Moreover, even though most participants were female caregivers, parents disclosed that they often shared with their husbands things that they learned during the program:

I share with my spouse things that I learnt. My husband also understands … meaning that when he sees that I am tired or came home late from [the] workplace, he will automatically help and does not complain. When I go home after the session, I will share with my husband. (35 years old, child group)

Respondents also reported improvements in relationships with their husbands after engaging in the program: “After the program, my communication with my husband improved” (35 years old, infant group). As another parent explained, her husband perceived the benefits of the program in terms of their relationship: “My husband does not like it if we are grumpy and short-tempered. Now that I am calmer, he is happy. He will say – it’s good this way, no more anger and scolding” (34 years old, infant group). On the other hand, one parent reported disagreements between her and her husband who did not support her attendance in the program, “My husband doesn’t quite like to look after our children … when I asked him to help look after children while I am attending the program, he will interrupt my session with phone calls … it is very stressful for me” (45 years old, adolescent group).

#### The need for additional further parenting assistance

Although parents reported positive impacts of Naungan Kasih, they also indicated areas in which they required additional assistance, especially regarding communication about sexuality and reproductive health and digital parenting. They suggested adding topics on sensitive and difficult topics to discuss with teenage children, such as sexuality and reproductive health. As one parent of an adolescent child expressed, "All these sensitive topics, how to handle them? For example, if our child tells us that she likes someone…like, 'Mum, I like this handsome boy,' What we are going to say?" (43 years old, adolescent group). Parents also noted that they lacked sufficient knowledge and skills in managing the use of cell phones and social media applications on cell phones and personal computers. Suggested topics included addressing cyberbullying and online sexual exploitation. Lastly, some parents remarked that they would appreciate more information and skills on how to manage children with different personalities: "Personality. Approach may not be the same. So how to deal with introvert and extrovert" (44 years old, child group).

### Adolescent respondents

**Fig. 6:**
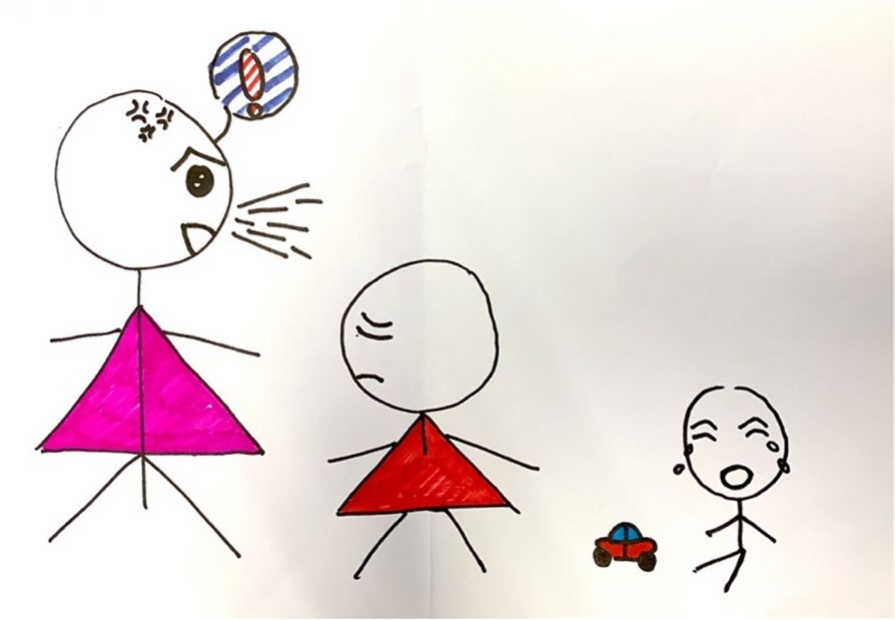
Adolescent drawing depicting the quality of their relationship with their parents before Naungan Kasih

Most adolescent respondents reported that their parents were often angry, high-spirited, and strict with them before the program. They shared that there was little positive communication between them and their parents and that they rarely spent time together. As one teen reported, “Everybody does their own business. Dad with his work while mum [is] busy with the younger kids. Everybody does their own things. I want to go out, but mum did not allow. Feeling being neglected” (16-year-old female). Some child respondents also reported that their parents primarily communicated to them by shouting and “nagging” (see Fig. 6 for illustration).

When asked about how this relationship had changed, most adolescents reported positive changes in their relationships with their parents. They shared that they became closer and more attached to their parents and are more open to communicating about personal issues. As one child stated, "When I have problems, mum will ask and talk to me. When I became quiet, she would ask why?" (16-year-old female). They also noted that their parents became more caring and understanding, they spent more time together and communicated positively and calmly with them. Another responded disclosed that, "We are closer to each other. If I share anything with her, she is responsive, give opinions" (13-year-old female). Children felt appreciated when parents spent more time with them during the program: “[Time together is the] best. Spending time together feels good” (13-year-old female). However, one teen respondent noted that he had observed little or no changes in the behavior of their mother’s behavior, “There are times she’s okay. Some other times, she scolds” (15-year-old male).

For the most part, adolescents also noticed that their parents were disciplining them in a more positive way. For example, one teenage reported that her mother was yelling and shouting less: “My mum approaches me nicely. No more scolding or raised voice. She has mellowed a bit” (17-year-old female). Many adolescents also shared that there was a difference in the way that their parents communicated with them even when they were being scolded. As one teen shared, "My mum now talks with lower tone voice, before this she was very fierce" (12-year-old female). In addition, many adolescents reported improvements in their own lives, including reduced stress after the Naungan Kasih program even though they did not attend sessions themselves. As one teen disclosed, “Previously I was quite stress, stressed about studies…. now less stressful. I used to keep my feelings and problems to myself, now [I] can share my feelings. I feel relieved” (17-year-old female). Teens also shared that these changes were primarily due to improved communication between themselves and their parents.

### Program facilitators

#### Improving skills over time

Facilitators expressed initial concerns about their capacity to deliver the parenting modules, as the collaborative and structured facilitation methods differed dramatically from their usual, more didactic method of instruction. However, over time they found that their confidence increased with practice:

At first, to do something that we have never done before, very structured, interrelated, must follow all the flow was quite tough for me. But after the second session, when rapport was already established, it was actually an enjoyable experience. (female, 36 years)

They identified specific factors that increased their confidence and competence. First, the structure of delivering the session with a co-facilitator provided opportunities to support to each other. As one facilitator expressed, “the facilitator and co-facilitator structure is good. We take turns so each of us can lead while the other write things down. We sort of complement each other. Sometimes I may forget something, and my partner will remind me. It helps to have that structure” (female, 39 years). Second, facilitators perceived the utility of using the House of Support model as a simple method of presenting the essential elements in the program as a guide, or map, for them to deliver the program. As another facilitator explained: “The whole process in the module is summarized in the House of Support. Everything is in that ‘house.’ If we truly comprehend the module, we can see where the process begins and ends … if we fail also, we can check where” (male, 39 years). Other respondents stated that the use of a flipchart to write ideas and opinions of parents helped make the content clearer and maintain focus during discussions. Furthermore, facilitators claimed that gender differences were not an issue in delivering the program, as male facilitators felt comfortable during delivery and perceived that they were well accepted by all the female participants in the group.

#### Challenges delivering the program

Facilitators mentioned several challenges experienced when delivering the program for the first time. Many reported that the additional responsibility made them feel burdened and overwhelmed with conflicting commitments to other work requirements within the LPPKN:

To me [delivering] the program is very tough, it is an additional burden to us, our normal jobs and tasks are the same if not more – on top of it we have to run the sessions. Very tiring, especially for people who live far from the location. There are some good parts about learning the new approach and what not but in general, it is burdensome. We hope there will be some recognition that we have done something extra, way above our normal duties. (female, 32 years)

One facilitator found it challenging to balance her role as a facilitator and a coordinator of program delivery. They also found it difficult to maintain their role as a group facilitator, given their previous experience as lecturers or counsellors. Facilitators also disclosed that they felt awkward when delivering content on the use of praise for positive reinforcement and on sexuality and reproductive health. For instance, one facilitator described her initial difficulty working with praise:

Some of the tasks that we need to teach we must do it in the session, such as we need to praise the participants too, so that one was not that easy because we are not used to praise people before this – just a bit awkward. But when we explain that what we learn in class is what to be done at home, the participants gradually accepted the idea as well. (female, 55 years)

Another facilitator stated that working with older caregivers and grandmothers was particularly challenging due to their resistance to change: “Handling the grandmothers was a bit challenging. I always felt uncertain about them not agreeing with whatever I said, the messages that we want them to learn etc. Because they are much older and “know more…that was the challenge for me” (male, 34 years). Finally, facilitators shared that they did not feel prepared to help participants manage inter-parental conflict affecting group dynamics.

When participants start sharing about how their husbands are not practicing good parenting even though they have shared what they learned in this program or when they have conflicts partly sound more like marital issues, I didn’t know how to respond to that because we were not trained on that subject during the training. [It’s] not covered in the module. (female, 32 years)

#### Training and support

Overall, facilitators reported that the 4-day training workshop was sufficient to prepare them for program delivery. However, they also identified several limitations. For instance, many of the facilitators found it difficult to participate fully in the workshop activities delivered in English by an international PLH trainer and found the level of translation by co-facilitators inadequate: “The language barrier was a bit challenging. To understand is one thing, to give feedback is the other. Even though there was [a] translation process, it is different from what was done in our language that we are more fluent with” (male, 34 years). They also explained that they needed to spend extra time studying the facilitator manual to prepare for program delivery: “We did not know that all that we learned during the trainings were exactly the things that we had to do in the program, until we really studied the module” (male, 39 years).

Facilitators overwhelmingly agreed that the supportive supervision by coaches from University Putra Malaysia during program delivery helped improve the quality of facilitation. They shared that the coaching increased their awareness of their limitations and provided opportunities to improve their skills. As one facilitator explained, “The coaching sessions give us opportunities to listen to feedbacks from the coaches, if we made mistakes, so sort of reminders for us” (male 34 years). They also expressed that the coaching motivated them to prepare for the next session and helped them identify ways to improve program delivery and coordination.

#### Suggested improvements

Facilitators also suggested several ways to improve the delivery of Naungan Kasih. They overwhelmingly supported the addition of content and training on sexuality and reproductive health, intimate partner relations, and online child protection. They also recommended that LPPKN managers made sure that the program is prioritized as part of their existing job descriptions and workplans. Additional suggestions included simplifying the manual in simpler terms and ensuring facilitators practiced delivering content before the group sessions. Furthermore, the facilitators agreed there were enough sessions for the parents, but that the content could be reduced in each session to increase the likelihood that they could deliver the entire session within two hours.

Facilitators also identified ways to improve training and support. It was suggested that future trainings are conducted in Bahasa or with professional interpreters. They also requested that workshops be delivered for smaller groups ranging from 10 to 15 trainees. Finally, facilitators recommended that future trainees receive program manuals prior to the training workshop.

### Harms

Apart for one participant reporting increased tension between herself and her husband who did not support her attendance, and the report of increased stress for facilitators due to increased workload, no significant harms were reported.

## Discussion

This study is the first of its kind in Malaysia to examine the feasibility of combining content from various evidence-based, social learning parenting programs to deliver a program for parents of children from birth to 17 years across the child development spectrum. Although results must be treated with caution due to the lack of a control group and small sample size, findings suggest that a universal program delivered by government staff on a community level may reduce some forms of violence against children in Malaysia. This expands current evidence from two randomized controlled trials in Malaysia, one which examined the effectiveness of a parenting program for parents of children aged 6 to 12 years with clinical levels of behavioral problems [33], and another delivered to Rohingya and Afghan refugees in Malaysia [34].

Quantitative analyses comparing parent-reported (all female caregivers) scores at post-intervention with those at baseline found less overall child maltreatment, less physical abuse, less emotional abuse, reduced attitudes supporting corporal punishment, less child behavior problems and improved attitude towards punishment. However, there were no reported changes on positive and harsh parenting, neglect, parental mental health, and marital satisfaction, nor were there any other significant changes from baseline to post-test assessments on the same outcomes when reported by children, though inferences for child-report are limited due to the small sample size.

Qualitative findings from in-depth interviews and focus groups suggest encouraging signs that the Naungan Kasih program had tangible benefits for female caregivers involved in the program. Respondents reported improvements in mental health, particularly stress and reactivity, that led to improvements in positive parent–child relationships. They also noticed positive changes in their children's behavior, including more open communication with their adolescent children, a finding that was corroborated by focus groups with adolescents. It is also promising that caregivers also reported improvements in their sense of parental self-efficacy and engagement. Furthermore, results indicating the program's benefits extended beyond the parent's target child to other children also suggests that the parenting skills may be applicable to a wider range of child ages. Lastly, even though the study only engaged female caregivers, there were some signs of spill-over benefits with husbands also learning about positive parenting skills from their partners. However, qualitative results also suggest that, for some families, the lack of male involvement may also have increased some inter-parental conflict over different parenting practices.

Although the structure, content, and delivery process were new to facilitators, many reported that their self-confidence with the program approach improved with practice. The Naungan Kasih structure of consecutive sessions delivered in a weekly series was a departure from normative practice for LPPKN programs which are normally delivered over intensive weekends. Nonetheless, qualitative and quantitative findings suggest that the format of delivery was acceptable to parents and facilitators. Despite initial reservations that parents would resist attending a program delivered over time, many facilitators noted the benefits of delivering the program over weekly sessions in which parenting skills are reinforced over time. Moreover, engagement data corresponds to findings from implementation studies on similar group-based parenting programs [35, 36]. The mean attendance rate was more than two-thirds of the program with over 60% of the sample attending five or more sessions. One might have expected even higher levels of engagement with a more selective sample since parents with children who have clinical levels of behavior problems are generally associated with greater attendance rates [37]. However, it is important to note that attendance was much higher in Shah Alam where parents were also from a higher socioeconomic background. More vulnerable families may require additional support to access in-person sessions, such as covering costs for transportation or offering alternative modes of engagement through hybrid and digital delivery [38, 39].

It is also worth noting the low concordance between adolescent report of program benefits in the outcome assessments (*n* = 26) compared to the qualitative focus groups (*n* = 12). Outcome assessments largely failed to show trends in the direction of benefit from the program, whereas the young people in the FGDs were very positive about the changes seen in family life, in line with the goals of the programs and the parents’ views. It is unclear whether this was an unrepresentative subgroup of respondents, or whether, overall, youth failed to see the same changes reported by parents who had received an intervention and therefore might be more susceptible to social desirability and reporting bias. These interpretations are necessarily made with great caution, given the very small number of child respondents in both quantitative and qualitative samples.

### Limitations

There were several limitations that require addressing. First, one must be cautious regarding the interpretation of pre-post results for several reasons, including a) self-report assessments being susceptible to social desirability and response bias, b) inability to examine potential sleeper or drop-off effects with longer-term follow-up assessments [40], c) lack of a comparison group and randomized design limiting an inference of causality, and d) small sample size with limited power to detect significant intervention effects. The latter was particularly true with the youth sample, though it also applies to the early childhood assessments. Future research would also benefit from examining clinical significance of results as well as reliable change indices to further understand the real-world implications of findings. In addition, it was challenging to use measurements that were applicable to the entire age range of children from birth to 17 years which required further division of the sample size into smaller groups. Future studies might benefit from either targeting a specific age range that is at a higher risk of maltreatment (e.g., young children ages 2–9 or adolescents ages 10–17) or by using more global assessments of parenting behaviors. Furthermore, since none of the measurements were previously validated in Bahasa Melayu or for Malaysian populations, it is possible that their psychometric properties were different than the original scales and thus did not accurately capture the intended outcomes. This is a much-needed area requiring additional research so that locally validated measurements may be used in subsequent studies.

Additionally, Naungan Kasih was delivered as a universal parenting program with limited screening for families with higher risks of abuse or dysfunctional relationships. There may have been a considerable proportion of parents who did not require intensive support but were rather simply interested in the program. In fact, systematic reviews have found lower program effectiveness of parenting programs delivered on a universal level in comparison to either selective prevention targeting families at higher risk or treatment for those with children with clinical levels of disruptive behavior or abusive parents [41]. Given the limited human and financial resources available, screening participants during recruitment may have been more efficient to identify more vulnerable families.

An additional limitation was the exclusive recruitment and engagement of female caregivers. While this is partly due to the recruitment and referral strategies employed during the study, the engagement of male caregivers is a common challenge for parenting programs on a global level [42]. Nonetheless, results are encouraging that male facilitators reported high level of confidence and acceptability when delivering the program to female caregivers despite predominant norms regarding gender in Malaysia [3]. Delivery for Muslim families may also require single-sex groups including separate male and female facilitators, though identifying ways for male and female caregivers to share experiences and discuss parenting principles may reduce potential inter-parental and intimate partner conflict [43].

## Conclusions

Results from the pilot study provide several areas for program revision that should be considered prior to further program delivery and scale-up of Naungan Kasih. First, findings suggest that additional training and support may help facilitators feel comfortable engaging parents in sensitive topics such as sexual and reproductive health. This was corroborated by parents who indicated that they would benefit from more content focused on adolescent sexuality and sexual behavior. Furthermore, facilitators and parents both indicated that families would have appreciated additional content on managing gadget use and online behavior. Likewise, the program lacked explicit attention to inclusivity, especially for parents and children living with disabilities. While parents with children living with disabilities may need specific content focused on addressing their needs [44], it was also suggested that the entire program undergo a disabilities review to make sure that the core content is also inclusive. In addition, program content may need to be adjusted when delivered to target populations such as Rohingya refugees [34]. Additional content is also necessary to address intimate partner relationships that also affect parent–child interaction. This should include more targeted male engagement to reduce the likelihood of inter-partner conflict. Gender transformative approaches may also increase male engagement in caregiving while supporting more equitable relationships [45]. Finally, LPPKN staff noted that the program needs to be integrated within their existing workflow to be sustainable both in terms of facilitator capacity and long-term scalability. Potential adaptations for digital and hybrid delivery may reduce the workload of facilitators while increasing the scalability of the program.

In conclusion, we note several key strengths of this study. First, it was designed from the outset in collaboration with the relevant government department. Second, despite the limitations of its pre-post design, it used a mixed-methods approach to examine the impact of the program on child and family outcomes include violence against children. Third, it triangulated data from multiple sources including parents, children, and facilitators. And finally, it addressed the need for evidence of effectiveness and implementation of universal parenting programs that can be delivered to parents of children from birth to 18 years within an existing community delivery system. Nonetheless, we strongly recommend further refinement, optimization, and testing prior to delivery at scale [46].

## Data Availability

The datasets generated and analysed during the current study are available in the Open Science Framework repository, (https://osf.io/c6ge8/).

## Abbreviations

APQ: Alabama Parenting Questionnaire
CASI: Computer assisted self-interviewing
CBCL: Child Behavior Checklist
DASS: Depression, Anxiety, and Stress Scale
FGD: Focus group discussion
ICAST: ISPCAN Child Abuse Screening Tool
IRR: Incidence risk ratio
LMIC: Low- and middle-income country
LPPKN: Lembaga Penduduk & Pembangunan Keluarga Negara (National Population and Family Development Board)
MICS: Multiple Indicator Cluster Survey
PLH: Parenting for Lifelong Health
VAC: Violence against Children
